# Households, the omitted level in contextual analysis: disentangling the relative influence of households and districts on the variation of BMI about two decades in Indonesia

**DOI:** 10.1186/s12939-016-0388-7

**Published:** 2016-07-07

**Authors:** Masoud Vaezghasemi, Nawi Ng, Malin Eriksson, S. V. Subramanian

**Affiliations:** Unit of Epidemiology and Global Health, Department of Public Health and Clinical Medicine, Umeå University, SE-901 87 Umeå, Sweden; Umeå Center for Global Health Research, Umeå University, Umeå, Sweden; Umeå Center for Gender Studies, Umeå University, Umeå, Sweden; Harvard Center for Population and Development Studies, Cambridge, MA USA; Department of Social and Behavioral Sciences, Harvard School of Public Health, Boston, MA USA

**Keywords:** Body mass index, Multilevel modelling, Omitted level, Contextual effect, Households, Indonesian family life survey

## Abstract

**Background:**

Most of the research investigating the effect of social context on individual health outcomes has interpreted context in terms of the residential environment. In these studies, individuals are nested within their neighbourhoods or communities, disregarding the intermediate household level that lies between individuals and their residential environment. Households are an important determinant of health yet they are rarely included at the contextual level in research examining association between body mass index (BMI) and the social determinants of health. In this study, our main aim was to provide a methodological demonstration of multilevel analysis, which disentangles the simultaneous effects of households and districts as well as their associated predictors on BMI over time.

**Methods:**

Using both two- and three-level multilevel analysis, we utilized data from all four cross-sections of the Indonesian Family life Survey (IFLS) 1993 to 2007-8.

**Results:**

We found that: (i) the variation in BMI attributable to districts decreased from 4.3 % in 1993 to 1.5 % in 1997-98, and remained constant until 2007–08, while there was an alarming increase in the variation of BMI attributable to households, from 10 % in 2000 to 15 % in 2007–08; (ii) ignoring the household level did not change the relative variance contribution of districts on BMI, but ignoring the district level resulted in overestimation of household effects, and (iii) households’ characteristics (socioeconomic status, size, and place of residence) did not attenuate the variation of BMI at the household-level.

**Conclusions:**

Estimating the relative importance of multiple social settings allows us to better understand and unpack the variation in clustered or hieratical data in order to make valid and robust inferences. Our findings will help direct investment of limited public health resources to the appropriate context in order to reduce health risk (variation in BMI) and promote population health.

## Background

During recent decades, a vast number of studies from epidemiology, sociology, human development and other disciplines have implied the important role played by context in a variety of health and developmental outcomes [1–8]. However, most research investigating the effect of context on individual health outcomes has mainly operationalized context within the area of residential environment, which generally refers to areas, neighbourhoods or communities. Consequently, individuals happen to be nested within their neighbourhoods or communities, ignoring the intermediate context that lies between individuals and their residential environment. Households or families as a major determinant of health [9, 10] can be considered in terms of an omitted contextual level, which has by and large been ignored in many empirical researches on social determinants of health. To a great extent, this is because of either simplicity or the absence of data at this level, which would allow the existence, and strength of such effects to be evaluated.

Few studies have examined the empirical implications or discussed the substantive eminence of this omitted level in empirical research [11–14]. The focus of these studies was mainly on methodological applications by using simulated data for analysis. We, however, examine the application of omitted level on health related outcomes – i.e. BMI – to better guide the limited public health resources to the right setting which has a greater impact on reducing variations in health among individual.

The reality recognized by social or behavioural scientists to understand social determinants of health and social disparity is largely multi-layered [15–18]. Assuming that individuals are nested in one and only one context may be an over-simplification of reality, as individuals simultaneously belong to multiple settings or levels that can each independently affect their health. Therefore, such a multilevel structure of reality means that empirical data sampled from individuals embedded in multiple social contexts are not mutually independent. Hence, in order to turn the complex models of social epidemiology into a useful analytical model of disease processes in persons and in populations, more comprehensive and better data are needed to test these models by using advanced statistical techniques and relevant epidemiological theories [19].

The empirical implications of an omitted level (in our example, households) can potentially alter the inferences drawn about the effect of the individual level or community level on a given outcome. It is possible that the clustering of the health of individuals within areas is due, in part, to the clustering of the health of individuals within households. Hence, studies that suggest the residential environment effects on individual health exist independently of individual characteristics need to be aware of the problem of ignoring households as a level in the multilevel analyses. Omission of this intermediate level can lead to biased parameter estimates and tests of cross-level effects and interactions, with potentially misleading substantive conclusions [20]. Consequently, the policy implication of such studies is that targeted interventions and policies are misguided to the wrong setting, whether it be the household, the neighbourhood, or both.

So far, there has been great effort to observe the trend—assessing the associated factors and explaining the variations—of ever-increasing mean body mass index (BMI) or the prevalence of overweight and obesity or the dual burden of malnutrition (the co-existence of under- and overweight individuals in the same household) in Indonesia [21–27]. Previous studies, however, analysed data either only at the individual level, using ordinary least regressions, or predominantly focused on neighbourhoods and districts by applying hierarchical two-level multilevel analyses of only one time point. Therefore, identification of a context capable of having the greatest effect on reducing disease risks and promoting population health has been missed. In this study, our main aim was to provide a methodical demonstration of three-level multilevel analysis to disentangle the simultaneous effect of households and districts and their associated predictors on BMI over time (from 1993 to 2007–8) in Indonesia.

The specific objectives of our study are: (i) to assess the extent to which variation in BMI is attributed to the individual level, household level and district level, and how it is changing over time; (ii) whether ignoring the household level results in over- or under-estimation of the relative variance contribution of the district; and (iii) to examine how much of the variation in BMI is explained by the characteristics of each level and how it is changing over time.

## Method

### Data source

We utilized a nationally representative data from an on-going longitudinal socioeconomic and health survey, called the Indonesian Family Life Survey (IFLS) (http://www.rand.org/labor/FLS/IFLS.html). So far, IFLS consists of four Waves from data collected in 1993, 1997–8, 2000, 2007–2008. The IFLS employed a multi-stage stratified systematic sampling design based on the stratification of provinces and urban/rural location. From 27 provinces, 13 of them were selected representing 83 % of the population. IFLS has a multilevel structure with individuals nested within households and districts [28]. It provides a rich set of information on individuals and households, the communities they live in, and the facilities available to them.

### Study subjects

**Fig. 1:**
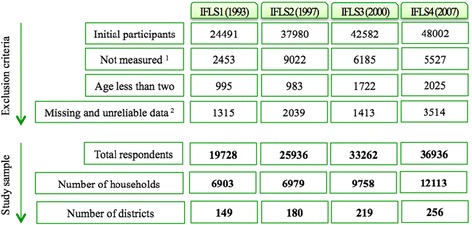
Study population, exclusion criteria, and the number of households and districts for each wave if Indonesian Family Life Surveys (IFLS). ^1^ Not measured is referred to those for whom anthropometric measurements were not available. The main reason for that was household members had moved to new households or a small number were not available for physical health measurements. ^2^ Among those with available anthropometric measurers, the exclusion criteria for the variables height, weight and BMI were (height < 100 & height > 200), (weight < 25 & weight > 200), and (8 > BMI > 45), respectively. IFLS: Indonesian Family Life Survey

We included all individuals in this analysis except children under two years old, pregnant women and individuals with missing values in all independent covariates. We also excluded individuals with extreme values of height (height <100 and height >200), weight (weight <25 and weight >200) and BMI (in Kg/m2: <8 and >45). After exclusion of outliers and missing values, a total number of 19,728 (Wave 1), 25,936 (Wave 2), 33,262 (Wave 3) and 36,936 (Wave 4) individuals were included in the analysis (Fig. 1). These individuals were nested in 6,903 households and 149 districts in Wave 1; 6,979 households and 180 districts in Wave 2; 9,758 households and 219 districts in Wave 3; and 12,113 households and 256 districts in Wave 4 (Fig. 1). Based on IFLS, a household was defined as a “group of people whose members reside in the same dwelling and share food from the same cooking pot” [29]. The number of excluded individuals in IFLS3 and IFLS4 was higher (about 17 and 16 %). Apart from having missing values and outliers in all covariates, a higher number of missing values was found in anthropometric measurements and the main reason was that household members had moved and a small number were not available for physical health measurements.

### Outcome variable

We calculated BMI (weight in kilograms divided by the square of height in meters) based on the World Health Organization (WHO) definition [30].

### Covariates

The covariates were: (i) sex (male and female); (ii) age (2–100 years); (iii) education (no schooling, elementary, secondary and university); (iv) occupation (never worked, worked, in school and retired); (v) marital status (never married, married, separated, divorced and widowed); (vi) household living standard presented as quartiles of per capita expenditure: the first quartile was considered the “lowest per capita expenditure”; (vii) household size; and (viii) place of residence (urban and rural). Household per capita expenditure calculated by IFLS was used as a proxy for a household’s living standard and contained information about the household’s food expenditure and non-food consumption during one month measured in Indonesian Rupiah [31]. Since our preliminary analysis showed only a very small percentage of variation in BMI was attributable to the district, district level variables were not included in the analysis.

### Analysis

In total, we utilized 36 multilevel (two-level and three-level) models to analyse the data. Nine multilevel models were run on each wave of IFLS. We started by fitting three sets of models in the current analysis. The first two models used the two-level multilevel model described in detail elsewhere [6, 20, 32]. These models assume a two-level multilevel data structure, where observations are hierarchically nested, such that members of the lower level of individuals (i.e. level one) are nested in one and only one entity at the higher level of households (i.e. level two). Thus, we began by fitting a two-level “household only” multilevel model (ignoring the districts), where the outcome (denoted y) for a person (denoted i) nested in a given household (denoted j) was modelled as:

1 $$ {y}_{ij} = {\ss}_0 + {\ss}_n{x}_{nij} + \left({u}_{0j} + {e}_{0ij}\right) $$

In Eq. (1), the fixed effect parameter *ß*_*0*_ refers to the overall mean outcome y across all households and *ß*_*n*_*x*_*nij*_ refers to a vector of individual level covariates. The random effect parameter *u*_*0j*_ refers to the random effect for the household (assumed to be normally distributed with a mean of 0 and variance *σ*^*2*^*u*_*0*_), and *e*_*0ij*_ refers to the random effect for the individual.

Secondly, we ran a two-level “district only” multilevel model (ignoring household), where the outcome (denoted y) for a person (denoted i) nested in a given district (denoted k) was modelled as:

2 $$ {y}_{ik} = {\ss}_0 + {\ss}_n{x}_{nik} + \left({u}_{0k} + {e}_{0ik}\right) $$

The fixed and random effect parameters in Eq. (2) have an identical interpretation to those in Eq. (1), except the y now refers to district instead of household. The ordinary least regression models, in which context is ignored, are not sufficiently adjustable to accommodate multiple nested contexts simultaneously, though the two-level multilevel modelling marks a significant advancement for considering context.

The third model we fitted was a three-level multilevel model in which individuals (denoted i) simultaneously belong to two nested contexts, here household (denoted j) and district (denoted k). Thus our outcome (denoted y) for a person (i) nested in a household (j) and district (k) is modelled as:

3 $$ {y}_{ijk} = {\ss}_0 + {\ss}_n{x}_{nijk} + \left({v}_{0k} + {u}_{0jk} + {e}_{0ijk}\right) $$

In Eq. (3), which presents a null or intercept-only model (i.e. a model without covariates), the fixed effect parameter *ß*_*0*_ refers to the overall mean outcome (y) across all households and districts; *v*_*0k*_ is the random effect at district level, and allowed to vary from the grand mean (variance between districts is assumed to be normally distributed with a mean of 0 and variance *σ*^*2*^*v*_*0*_); *u*_*0jk*_ is the random effect at the household level, a departure from the household effect within the district level (variance between households is assumed to be normally distributed with a mean of 0 and variance *σ*^*2*^*u*_*0*_); *e*_*0ijk*_ is the random effect at the individual level, a departure from the household effect within a district (variance between individuals is assumed to be normally distributed with a mean of 0 and variance *σ*^*2*^*e*_*0*_).

We continued the analyses in three steps on all four IFLS waves. First, to partition the variance in BMI into within and between components and estimate an intra-class correlation coefficient (ICC)—i.e. the proportion of variation in the outcome that was due to differences across households and districts, rather than differences across individuals—we estimated Model 1 controlling for only age and sex. The ICCs in the household-only and district-only multilevel model were generated by dividing the between-level random effect by the total variance. In the three-level multilevel analysis, we calculated ICCs for district-level and household-level, which are referred to as the intra-district (i.e. correlation in outcome between two individuals who live in the same district but live in different households; this was calculated by dividing the district-level random effect by the total variance, or the sum of the three variance components) and intra-household correlation coefficient (i.e. correlation in outcome between two individuals who live in the same household; this was calculated by dividing the household-level random effect by the total variance). Subsequently, we estimated a model that contained other individual-level predictors and covariates (Model 2). By including these individual-level variables, we were able to evaluate the extent to which the between-level variance estimates (i.e. random effect parameters) could be explained by the observed individual characteristics across households and districts. We then fitted a model containing individual-level variables and household-level variables (Model 3).

For each wave of IFLS, we examined residual plots at each level of analysis to evaluate model diagnostics on the variance parameter; this enabled us to test model assumptions, and detect outliers and influence points on model fit. Analyses were conducted using unweighted data; however, a non-weighted analysis is also appropriate as our emphasis was on tests of association and random effects, rather than deriving nationally representative estimates, and we adjusted our analyses for sample characteristics and thus reduced the heterogeneity in the sample [33]. All analyses were conducted by STATA version 14.1 (College Station, Texas 77845, USA).

## Results

### Descriptive and demographic characteristics of the study population

We analysed data from 19,728 (Wave 1), 25,936 (Wave 2), 33,262 (Wave 3) and 36,936 (Wave 4) individuals. These individuals were nested in 6,903 households and 149 districts in Wave 1; 6,979 households and 180 districts in Wave 2; 9,758 households and 219 districts in Wave 3; and 12,113 households and 256 districts in Wave 4 (Fig. 1). In order to clarify whether households’ basic demographic composition characteristics had substantially changed over time (1993–2007), for each study cycle we estimated mean household size and its standard deviation (SD): for IFLS1 4.0 (1.5); for IFLS2 6.5 (2.6); for IFLS3 5.5 (2.3); and for IFLS4 4.5 (1.9), mean household age and its SD: for IFLS1 29.2 (14.2); for IFLS2 22.9 (9.7); for IFLS3 25.6 (10.8); for IFLS4 29.4 (18.7), and also proportion of men versus women: for IFLS1 45.7 (19.3); for IFLS2 38.0 (20.0); for IFLS3 42,6 (22.1); for IFLS4 47.4 (19.8) (Table 1). The results from Table 1 indicate that even though there was a large variation within each wave because of large SD, the composition of households remained relatively similar across the four study cycles. In addition, BMI and SD increased in all age groups but the increase was more prominent among the middle age groups (30–70).

**Table 1:** Individuals’ and households’ basic demographic composition characteristics. Indonesian Family Life Survey (IFLS) 1993–2007

		IFLS1	IFLS2	IFLS3	IFLS4
		Mean (sd)	Mean (sd)	Mean (sd)	Mean (sd)
BMI	Age 2–10	15.0 (2.03)	15.0 (2.12)	15.0 (2.12)	15.4 (2.68)
	Age 11–20	17.8 (3.18)	18.7 (3.01)	18.8 (3.01)	19.0 (3.40)
	Age 21–30	21.3 (2.83)	21.2 (3.15)	21.2 (3.15)	22.0 (3.73)
	Age 31–40	22.0 (3.30)	22.3 (3.63)	22.5 (3.63)	23.3 (4.07)
	Age 41–50	21.9 (3.59)	22.6 (3.90)	22.9 (3.99)	23.7 (4.22)
	Age 51–60	20.9 (3.76)	21.4 (3.79)	21.8 (4.08)	23.1 (4.33)
	Age 61–70	20.4 (3.79)	20.6 (3.87)	20.7 (3.86)	21.5 (4.08)
	Age 71–80	19.4 (3.21)	19.6 (3.55)	19.8 (3.65)	20.5 (3.94)
	Age 81–90	19.0 (2.49)	19.5 (3.31)	19.3 (3.69)	19.9 (3.27)
	Age 91–100	22.0 (3.84)	19.4 (3.01)	18.9 (2.93)	19.5 (2.36)
Household size		4.0 (1.5)	6.5 (2.6)	5.5 (2.3)	4.5 (1.9)
Household age		29.2 (14.2)	22.9 (9.7)	25.6 (10.8)	29.4 (18.7)
Percentage of Men		45.7 (19.3)	38.0 (20.0)	42.6 (22.1)	47.4 (19.8)

### Average BMI distribution across households and districts

**Fig. 2:**
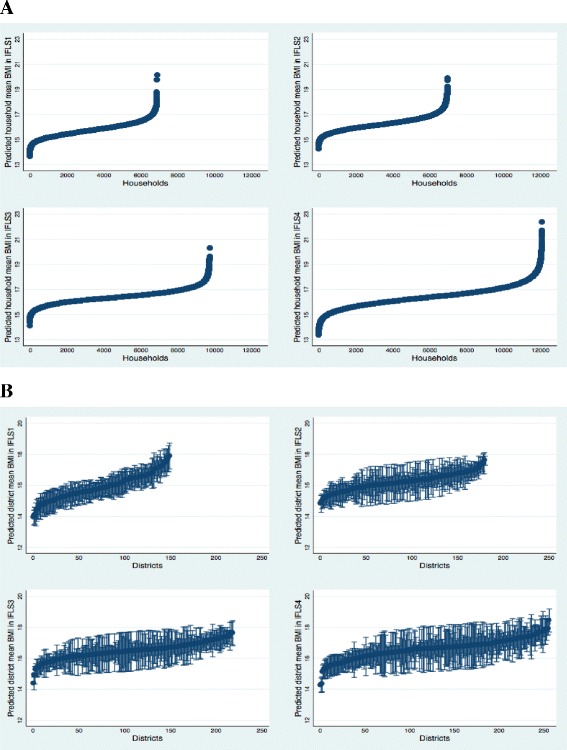
Distribution of BMI mean within and between **a** households and **b** neighborhoods. Indonesian Family life survey (IFLS) 1993–2007. Note. Dots represent the mean within-household and within-district BMI mean. Ninety-five percent bound around the means (based on the standard deviations of BMI mean); these values are excluded for households owing to the high number of households present in the data. Values are sorted from left to right by lowest household or district mean

After controlling for age and sex (Model 1), the average predicted BMI (household mean = 15.7, SD = 3.4: district mean = 15.9, SD = 3.5 in IFLS1; household mean = 16.2, SD = 3.4: district mean = 16.2, SD = 3.6 in IFLS2; household mean = 16.4, SD = 3.6: district mean = 16.4, SD = 3.7 in IFLS3; household mean = 16.4, SD = 3.8: district mean = 16.5, SD = 4.1 in IFLS4) was rather similar across households and districts. However, the standard deviation of average BMI had increased for both households and districts over time. In addition, as shown in Fig. 2, there was considerable variability within and between these contexts with respect to average BMI. For example, in IFLS4, the mean BMI varied from 13.3 to 22.4 between households and from 14.5 to 18.8 between districts (Fig. 2). Thus, the distribution of BMI was not uniform, but rather varied as a result of the household or district context.

### Multilevel modelling and model comparison

Table 2 presents the results of a series of models (Model 1, Model 2 and Model 3) for the household-only model (two-level), district-only model (two-level), and household/district model (three-level), predicting BMI over time. In the first model, we controlled only for individuals’ age and sex.

**Table 2:** Random effect estimates at the household- and district-level for the household-only two-level multilevel model, district-only two-level multilevel model, and household/district three level multilevel model. Indonesian Family life survey (IFLS) 1993–2007

		ONLY HOUSEHOLD MODEL (TWO-LEVEL)				ONLY DISTRICT MODEL (TWO-LEVEL)				HOUSEHOLD AND DISTRICT MODEL (THREE-LEVEL)			
		IFLS1	IFLS2	IFLS3	IFLS4	IFLS1	IFLS2	IFLS3	IFLS4	IFLS1	IFLS2	IFLS3	IFLS4
		Est. (SE)	Est. (SE)	Est. (SE)	Est. (SE)	Est. (SE)	Est. (SE)	Est. (SE)	Est. (SE)	Est. (SE)	Est. (SE)	Est. (SE)	Est. (SE)
Model 1	Individual (Var)	11.534 (0.146)	11.822 (0.119)	12.573 (0.112)	14.703 (0.130)	12.338 (0.125)	12.723 (0.112)	13.584 (0.105)	16.526 (0.122)	11.544 (0.145)	11.780 (0.119)	12.590 (0.112)	14.660 (0.129)
	Household (Var)	1.500 (0.117)	1.343 (0.083)	1.408 (0.077)	2.426 (0.105)					0.821 (0.103)	0.970 (0.077)	1.004 (0.071)	1.911 (0.097)
	District (Var)					0.773 (0.085)	0.438 (0.064)	0.394 (0.054)	0.605 (0.075)	0.769 (0.109)	0.426 (0.065)	0.404 (0.059)	0.592 (0.077)
	Household (ICC)	0.115 (0.008)	0.102 (0.006)	0.101 (0.005)	0.142 (0.006)					0.063 (0.004)	0.073 (0.004)	0.072 (0.006)	0.111 (0.010)
	District (ICC)					0.059 (0.008)	0.0333 (0.005)	0.028 (0.004)	0.035 (0.004)	0.058 (0.008)	0.032 (0.005)	0.029 (0.004)	0.034 (0.004)
	Household/District (ICC)									0.121 (0.011)	0.106 (0.007)	0.101 (0.006)	0.146 (0.006)
Model 2	Individual (Var)	7.777 (0.098)	8.348 (0.084)	9.092 (0.081)	10.786 (0.095)	8.761 (0.088)	9.315 (0.082)	10.75 (0.078)	12.778 (0.094)	7.758 (0.097)	8.325 (0.084)	9.059 (0.081)	10.760 (0.095)
	Household (Var)	1.472 (0.085)	1.221 (0.064)	1.178 (0.058)	2.382 (0.084)					1.038 (0.077)	1.010 (0.059)	1.020 (0.055)	2.067 (0.080)
	District (Var)					0.553 (0.077)	0.248 (0.037)	0.211 (0.032)	0.389 (0.050)	0.547 (0.079)	0.250 (0.040)	0.215 (0.035)	0.367 (0.050)
	Household (ICC)	0.159 (0.008)	0.128 (0.006)	0.115 (0.005)	0.181 (0.006)					0.111 (0.009)	0.105 (0.008)	0.099 (0.006)	0.157 (0.008)
	District (ICC)					0.059 (0.008)	0.026 (0.004)	0.020 (0.003)	0.029 (0.004)	0.058 (0.008)	0.026 (0.004)	0.021 (0.003)	0.028 (0.004)
	Household/District (ICC)									0.170 (0.010)	0.131 (0.007)	0.120 (0.006)	0.184 (0.006)
Model 3	Individual (Var)	7.731 (0.097)	8.226 (0.083)	8.959 (0.080)	10.739 (0.094)	8.698 (0.088)	9.219 (0.081)	9.936 (0.077)	12.621 (0.093)	7.746 (0.097)	8.219 (0.083)	8.949 (0.080)	10.735 (0.094)
	Household (Var)	1.316 (0.080)	1.139 (0.060)	1.113 (0.057)	2.104 (0.079)					0.990 (0.075)	1.026 (0.059)	1.004 (0.055)	1.926 (0.077)
	District (Var)					0.416 (0.061)	0.158 (0.028)	0.162 (0.028)	0.244 (0.036)	0.391 (0.061)	0.140 (0.028)	0.145 (0.027)	0.208 (0.034)
	Household (ICC)	0.145 (0.008)	0.122 (0.006)	0.110 (0.005)	0.164 (0.006)					0.108 (0.008)	0.109 (0.006)	0.099 (0.005)	0.150 (0.006)
	District (ICC)					0.045 (0.006)	0.017 (0.003)	0.016 (0.003)	0.019 (0.003)	0.043 (0.006)	0.015 (0.003)	0.014 (0.003)	0.016 (0.003)
	Household/District (ICC)									0.151 (0.009)	0.124 (0.006)	0.114 (0.006)	0.166 (0.006)

*ICC* interclass correlation

Model 1: *age sex;* Model 2: *Model1 + education + marital status + occupation;* Model 3: *Model2 + households per-capita-expenditure + households size + urban/rural*

#### Changing attributable variance of households and districts over time

**Fig. 3:**
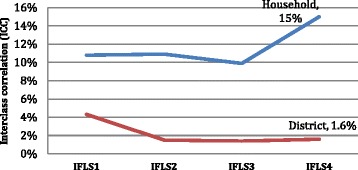
Household and district interclass correlation (ICC) of Body Mass Index (BMI) over four cycles of Indonesian Family Life Survey (IFLS)

We observed how much of the variation in BMI is attributable to the household and district level and how it is changing over time. Figure 3, Model 3 illustrates that from 1993 in IFLS1 to 2000 in IFLS3, about ten per cent of the variation of BMI was attributable to household-level characteristics; however, a sharp increase was found in 2007 in IFLS4, where 15 % of the variation in BMI was due to differences between households. In total contrast, the variation of BMI attributable to district characteristics decreased from 4.3 % in IFLS1 to 1.5, 1.4 and 1.6 % in IFLS2, IFLS3 and IFLS4 respectively (Fig. 3, Model 3).

#### Ignoring intermediate (household) level—a comparison between district only model (two-level) and household/district model (three-level)

In all three models (Model 1, Model 2 and Model 3), the random effects for district (*σ*^*2*^_*u0j*_) and district level ICC did not change from the district only model (two-level) to the household/district model (three-level). For instance, comparing the district only model (two-level) to the household/district model (three-level) for Model 3, the district level ICC remained almost the same from 4.5 to 4.3 % for IFLS1; 1.7 to 1.5 % for IFLS2; 1.6 to 1.4 % for IFLS3; and 1.9 to 1.6 % for IFLS4. This result suggests that if we ignore intermediate (household) level, we will neither overestimate nor underestimate the effect of higher (district) level.

#### Ignoring higher (district) level—a comparison between household only model (two-level) and household and district model (three-level)

In all three models (Model 1, Model 2 and Model 3), the random effects for household (*σ*^*2*^_*u0j*_) and household level ICC decreased from the household only model (two-level) to the household/district model (three-level) for each study wave. For instance, comparing the household only model (two-level) to the household/district model (three-level) for Model 3, the household level ICC decreased from 14.5 to 10.8 % for IFLS1; from 12.2 to 10.9 % for IFLS2; from 11.0 to 9.9 % for IFLS3; and from 16.4 to 15 % for IFLS4. This result suggests that if we ignore the higher (district) level, we will overestimate the effect of intermediate (household) level.

#### Explained variance by individuals and household characteristics—a comparison of Model 1, Model 2 and Model 3

We also found that inclusion of individual characteristics (education, marital status and occupation) in Model 2 largely attenuated the individual residual variance and between-level variance for districts in all four IFLS waves and in all three (household only, district only and household/district) models. This decline suggests that the between-level variation in BMI was due to compositional effect (i.e. characteristics of individuals in these contexts). However, compared to Model 2, inclusion of households’ characteristics (households’ per capita expenditure, households’ size and place of residency) in Model 3 did not reduce the residual variance at household level. For example, in the household/district model (three-level), the household residual variance in Model 2 was 1.04 (IFLS1), 1.01 (IFLS2), 1.02 (IFLS3) and 2.07 (IFLS4), which remained very similar in Model 3: 0.99 (IFLS1), 1.03 (IFLS2), 1.00 (IFLS3) and 1.93 (IFLS4). This comparison suggests that households’ per capita expenditure, size and place of residency could not explain or reduce the variation at household level.

## Discussion

The main findings in our study were: (i) a greater increase in the variation of BMI attributable to the households, compared with the districts; (ii) ignoring the household-level did not change the relative variance contribution of the districts on BMI, but ignoring the district-level resulted in overestimation of the household effect; (iii) households’ socio-economic characteristics did not attenuate BMI variation at the household-level.

Our study has uncovered one evidently very relevant context—households, whose study has remained almost quiescent in multilevel studies endeavouring to explain the fast growing body mass index in Indonesia. We focused on the significance of district and household factors in explaining variation in BMI over time and the results showed the salient relative importance of household over district. The variation in BMI attributable to districts decreased from 4.3 % in 1993 to 1.5 % in 1997–98 and remained constant until 2007–08, while there was a sharp increase in the variation of BMI attributable to households from 10 % in 2000 to 15 % in 2007–08. However, one must be cautious in stating that household level factors in general are more important for explaining health variation or inequality compared to factors at the local area/district level. It might well be that the influence of various contexts differs due to the particular health outcome in focus. One of the strongest predictors for BMI is food consumption [34], an event that takes place mainly within households. It is now evident that food consumption patterns are undergoing substantial changes in LMICs as well as in Indonesia, moving away from traditional cereals toward higher value and higher protein foods [35, 36]. Meals are planned, prepared and shared within the household and therefore there are strong reasons to believe that household factors are more important in influencing food intake compared to more distal factors such as the physical and social environment in the district. Several studies have shown how inhibited overeating is related to adult overweight [37], while children in general remain the highest proportion of underweight individuals [26] causing even more variation within the households. Our finding is in line with a study from Roemling and Qaim [25], who revealed increasing intra-household nutritional inequality in Indonesia. Indonesia has been through different economic stages: before economic crisis (prior to 1993), during economic crisis (1997–98), during economic recovery and decentralization (2000–2005) and in the phase of economic improvement (2006 onwards). The increased inequality in BMI within the households could be due to different impacts of the nutrition transition on different age groups, which affects the nutritional status of children and adults inversely. In contrast, lifestyle and dietary changes combined with limited nutrition and health knowledge on the one hand, coupled with rapid social, economic and technological changes on the other hand, seem be similarly shared within the districts among individuals after decentralization and during the phase of economic improvement. Therefore, this secular trend might be explained by sharing the same obesogenic environment at the district level.

However, beyond BMI, there are probably other health related outcomes where distal factors in the living environment might be more influential, such as self-rated health, for example. Within the research field of social capital and health, there is now quite strong evidence, based on multilevel studies, for a positive association between self-rated health and residential environment (community/neighbourhood/state/nation) characterized as high in social capital (i.e. socially cohesive, supportive and trusting) [38–40]. One may argue that household composition could contribute to the observed larger variation in BMI in 2007 as compared to 2000, since the number of households interviewed in 2007 increased by 30 % compared to those in 2000: it is likely that these new split-off households are possibly younger adults with children, and this can therefore contribute to a larger variation in the distribution of BMI at the household level. We examined the proportion of all individuals in each age category at ten year intervals. Even though the number of individuals and new split-off households increased over time, there was no substantial difference in proportion between under 20 year olds and those aged over 80 (data not shown). It should also be noted that there is still district level inequality in BMI (ICC = 1.6 % in 2007–08). These geographical variations in overweight and underweight individuals were also observed in a study by Hanandita and Tampubolon (2015) in Indonesia using data from Riskesdas, which covers more islands of the archipelago such as Sulawesi, Maluku, Halmahera, Nusa Tenggara and Papua. Nevertheless, the variation in districts did not increase over time as it did in households.

Apart from the substantive importance of households, the empirical applications of households as an omitted level are also important in contextual analysis. The notion of an omitted intermediate level and the importance of simultaneous analysis of the effect of multiple settings are more prominent where individuals are concurrently nested within multiple non-hierarchical settings—i.e. schools and neighbourhoods. Applying cross-classified multilevel models, these studies repeatedly reported a substantial decline in the role of neighbourhoods in children’s health outcomes after inclusion of the intermediate level such as schools [41–44]. In other words, ignoring an intermediate level (i.e. school) will result in overestimating the higher level (i.e. neighbourhood) where the structure of the data is not hierarchical. In our analysis, we examined the BMI of individuals who are nested within multiple hierarchical settings—household and districts—by applying three-level multilevel analysis. After accounting for the simultaneous effect of households and districts compared with district only models, there was no evidence as to whether the influence of the higher level (districts) is under- or overestimated. Nevertheless, had we estimated only a district level multilevel model and not estimated a three-level multilevel model that accounted for the random effect of both households and districts, we would have missed the contribution of households (ICC = 15 %) in the variation of BMI which would have remained otherwise unexplained. Furthermore, we now know the salient relative importance of households over districts, yet the answer to the question of *why* households became so different over time remains understudied.

A few methodological studies have investigated the effect of ignoring a level in the hierarchical nesting multilevel structure on test statistics using mainly simulated data [12, 13, 45–47]. These studies reported that when the higher level nesting structure is ignored, the variance of the ignored higher level relocates to the adjacent level and the variance component at the lowest level remains unchanged [12, 13, 45–47]. This is in line with our analysis when we compared the two-level (household only) model to the three-level (both household and district) model. In other words, the effect of household level is overestimated if the district level is ignored. The various authors also reported that if the intermediate level is ignored, the variance estimate of the discarded level is divided between the flanking levels and will be largely relocated to the lower level [12, 13, 45–47]. Especially when the data are perfectly balanced, the higher level variance will remain the same, but the lower level variance will now be subdivided between the intermediate and lower levels; however, the more unbalanced the data, the more approximate the estimates will become. This is also in accordance with our findings when we compared the two-level (district only) model with three-level (both household and district) model. Therefore, it can be concluded that the district level effect is neither over- nor underestimated when household level is ignored, while it is the variation at the individual level, which is overestimated.

Although fixed effect estimates (mean-centric measures) are informative for determining the extent to which the predictor of interest is associated with the outcome and also the degree to which it reduces between-level variation, in this analysis we purposely focused on only the random effect (measures of variance) of the multilevel contextual modelling. One reason for doing this was that we ran 36 multilevel models on all four cross-sections of IFLS data; therefore, reporting associations for a large number of predictors would have encumbered the current study. Furthermore, as George A. Kaplan put it in a paper called ‘What is wrong with social epidemiology, and how can we make it better’, many studies stop at reporting a statistically significant association between the risk factor and outcomes in question after adjusting for a set of known predictors [19]. This counteracts the detection of new risk factors, which could possibly motivate the search for new disease mechanisms and ascertain new social risk factors as something more informative. Juan Merlo et.al. (2009) proposed the variance approach (random effect) as a new approach to contextual analysis allowing perspectives to be explained which could not be interpreted in mean-centric (fixed effect) terms [48]. In our analysis, we compared the variance contributions (i.e. the random effect) across models to evaluate the extent to which inclusion of household predictors helped to explain the observed between-household variation in BMI. The associations between almost all of these fixed effect variables and BMI were statistically significant (data not shown). However, as reported earlier, it has not resulted in reducing the between-level variance. We may consider these factors to be significantly associated with BMI, but identifying the mechanism under which these associations are explained is awkward. Geoffrey Rose (1992) claims that “the primary determinants of disease are mainly economic and social, and therefore its remedies must also be economic and social” [49]. If these socioeconomic factors cannot or do not explain within-household variation in BMI within the 1993–2007 period in Indonesia, our findings beg the question of what other unknown household characteristics—such as family psychosocial conditions, family social capital, social network, gendered power relations, household decision making or women’s education—could have a larger effect on reducing the variation of BMI at the household level. Therefore, the need to unpack further the nature of within-household behaviour to explain the variation in BMI is a necessity.

Our study has some limitations that should be mentioned. First, the last wave of the IFLS was conducted in 2007–08, which might not be very up to date. However, IFLS is the largest longitudinal nationally representative survey, providing comprehensive socioeconomic and health information at the individual, household, district and province levels in Indonesia. Secondly, individuals who moved out and started a new split-off household in the subsequent waves were followed over time. Therefore, in IFLS2, IFLS3 and IFLS4, we had a smaller number of individuals in some districts. In practice, however, the assumption of a completely balanced design is almost never met [45]. In addition, referring to the concept of exchangeability in hierarchical models [50], each parameter borrows strength from the other parameters at its level in the hierarchy: therefore estimates are shrunk towards the population mean. The consequence of exchangeability is beneficial when the number of individuals observed in some of the units in the hierarchy is very small. Finally, we did not include the dietary pattern of households or the physical activity level of individuals, which might better explain the variation in BMI.

## Conclusion

Estimating the effect of the omitted level would help better to understand and unpack the variation in clustered or hierarchical data in order to make a valid and robust inference. The questions raised in our analysis provide a compelling agenda for further investigation where little is known about the consequence of ignoring a level in nesting multilevel models on different health-related outcomes. Recognition of increased variation in BMI at household level is important for laying out strategies that respond to the differential needs of individuals within the same household. Hence, to help guide the investment of limited public health resources, much more work is needed in this area to demonstrate and to evaluate the role of social context on health. Otherwise, implementation of misguided policies and interventions in contexts that may not be capable of having a significant effect on reducing health risk and promoting population health outcomes will always be a risk.

## Abbreviations

BMI, body mass index; ICC, intraclass correlation; IFLS, Indonesian Family Life Survey; LMICs, Low- and middle-income countries; SD, standard deviation; SES, socioeconomic status.
